# RNA-seq analysis and gene expression dynamics in the salivary glands of the argasid tick *Ornithodoros erraticus* along the trophogonic cycle

**DOI:** 10.1186/s13071-021-04671-z

**Published:** 2021-03-20

**Authors:** Ricardo Pérez-Sánchez, Ángel Carnero-Morán, Beatriz Soriano, Carlos Llorens, Ana Oleaga

**Affiliations:** 1 Parasitología Animal, Instituto de Recursos Naturales y Agrobiología de Salamanca (IRNASA, CSIC), Cordel de Merinas, 40-52, 37008 Salamanca, Spain; 2 Biotechvana, Scientific Park, University of Valencia, Calle Catedrático José Beltrán 2, Paterna, 46980 Valencia, Spain

**Keywords:** *Ornithodoros erraticus*, Soft ticks, Salivary glands, Transcriptome, Differential gene expression, Vaccinomics, Vaccines

## Abstract

**Background:**

The argasid tick *Ornithodoros erraticus* is the main vector of tick-borne human relapsing fever (TBRF) and African swine fever (ASF) in the Mediterranean Basin. Tick salivary proteins secreted to the host at the feeding interface play critical roles for tick feeding and may contribute to host infection by tick-borne pathogens; accordingly, these proteins represent interesting antigen targets for the development of vaccines aimed at the control and prevention of tick infestations and tick-borne diseases.

**Methods:**

To identify these proteins, the transcriptome of the salivary glands of *O. erraticus* was *de novo* assembled and the salivary gene expression dynamics assessed throughout the trophogonic cycle using Illumina sequencing. The genes differentially upregulated after feeding were selected and discussed as potential antigen candidates for tick vaccines.

**Results:**

Transcriptome assembly resulted in 22,007 transcripts and 18,961 annotated transcripts, which represent 86.15% of annotation success. Most salivary gene expression took place during the first 7 days after feeding (2088 upregulated transcripts), while only a few genes (122 upregulated transcripts) were differentially expressed from day 7 post-feeding onwards. The protein families more abundantly overrepresented after feeding were lipocalins, acid and basic tail proteins, proteases (particularly metalloproteases), protease inhibitors, secreted phospholipases A2, 5′-nucleotidases/apyrases and heme-binding vitellogenin-like proteins. All of them are functionally related to blood ingestion and regulation of host defensive responses, so they can be interesting candidate protective antigens for vaccines.

**Conclusions:**

The *O. erraticus* sialotranscriptome contains thousands of protein coding sequences—many of them belonging to large conserved multigene protein families—and shows a complexity and functional redundancy similar to those observed in the sialomes of other argasid and ixodid tick species. This high functional redundancy emphasises the need for developing multiantigenic tick vaccines to reach full protection. This research provides a set of promising candidate antigens for the development of vaccines for the control of *O. erraticus* infestations and prevention of tick-borne diseases of public and veterinary health relevance, such as TBRF and ASF. Additionally, this transcriptome constitutes a valuable reference database for proteomics studies of the saliva and salivary glands of *O. erraticus*. 
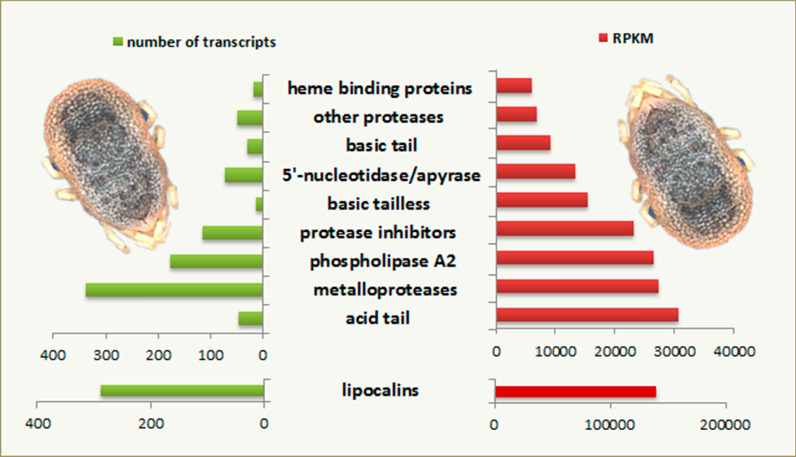

**Supplementary Information:**

The online version contains supplementary material available at 10.1186/s13071-021-04671-z.

## Background

Ticks are hematophagous ectoparasites of medical and veterinary importance worldwide because they cause direct injury to their hosts and transmit a large range of pathogens that affect wild and domestic animals, pets and humans, causing significant financial losses globally [1, 2]. There are two main tick families, Ixodidae (hard ticks) and Argasidae (soft ticks), which differ in important morphological and biological traits. Typically, ixodids are exophilic ticks that stand on soil and vegetation questing for suitable hosts. They spend several days feeding on the host and ingest large amounts of blood; once fed, the immature stages moult to the following stage and the adult ticks reproduce and die. By contrast, argasids typically are endophilic parasites that live inside the burrows of their wild hosts, domestic animal facilities and human houses. Argasids are fast-feeding ticks that complete their blood meal in < 1 h and, after engorgement, moult and reproduce inside their shelters. Adult argasids can repeat this trophogonic cycle up to ten times [3, 4].

The argasid tick *Ornithodoros erraticus* is distributed throughout the Iberian Peninsula, north and western Africa and western Asia [5]. This tick represents a medical and veterinary concern because it is the main vector in the Mediterranean Basin of the African swine fever (ASF) virus and of several *Borrelia spp.* spirochetes that cause tick-borne human relapsing fever (TBRF) [6, 7]. *Ornithodoros erraticus* is also the type species of the “*O. erraticus* complex,” and several species in this complex, such as *O. asperus*, *O. lahorensis*, *O. tartakovsky* and *O. tholozani,* are distributed throughout the Caucasus, the Russian Federation, the Middle and the Far East, where they transmit diverse species of TBRF-causing borreliae [1, 8, 9]. Furthermore, the ASF virus has spread out of control throughout this area in the last 10 years [10–13]; although not yet experimentally confirmed, if these tick species belonging to the “*O. erraticus* complex” are also competent vectors of the ASF virus, then their presence in anthropic environments would significantly increase transmission and long-term persistence of ASF in this vast region. Thus, effective prevention and control of TBRF and ASF in the affected areas would require the elimination of *Ornithodoros* vector populations from at least the anthropic environments.

Tick control mainly relies on application of chemical acaricides, but this selects resistant tick strains and accumulates chemical residues in animal products and the environment [14]. Moreover, the efficacy of acaricide application against *Ornithodoros* ticks is seriously limited because acaricides do not reach these parasites inside their shelters [15]. Thus, alternative methods for the control of ticks are urgently needed and immunological control is the most promising, environmentally friendly and sustainable strategy [16–18].

Success in research for anti-tick vaccine development largely depends on the identification of tick protective antigens. Despite the remarkable efforts that have been invested in the last 2 decades to identify and characterise antigen candidates for tick vaccines, only a limited number of partially protective tick antigens are currently available, mostly from ixodids and less from argasids [19–22].

Searching for and identifying new protective tick antigens are being currently approached by the selection of candidate protective antigens that have important biological functions, particularly among the molecules and biological processes specifically evolved by ticks to adapt to a strict haematophagous lifestyle [23–26], namely, the processes that are involved in host attachment, blood ingestion and modulation of the host defensive responses and are carried out by salivary proteins secreted into the host by tick saliva [27–29]. Also involved are the processes related to blood digestion, including nutrient transport and metabolism, management of iron and heme groups, detoxification and oxidative stress responses, which are accomplished by proteins expressed in the midgut [30–35].

Accordingly, the salivary glands and midguts of an increasing number of tick species have been subjected to transcriptomics and proteomics analyses and the resulting transcriptomes and proteomes of salivary glands (sialomes) and midguts (mialomes) have been annotated and inspected in vaccinomics pipelines for the selection and characterisation of candidate protective antigens [21, 36].

Most of the studies involving salivary glands have been performed on ixodids and to a lesser extent on argasids. As a result, the sialomes of 26 ixodid and six argasid ticks, including *Ornithodoros coriaceus, Ornithodoros parkeri, Ornithodoros turicata, Ornithodoros rostratus, Argas monolakensis* and *Antricola delacruzi*, have already been published [37–42]. Additionally, the sialotranscriptome of the argasid tick *Ornithodoros moubata* has recently been obtained in a study analogous to the present one [43]. These tick sialomes have revealed thousands of protein-coding sequences and large multigene protein families, many of them conserved between both tick families, reflecting high complexity and functional redundancy in the composition of tick sialomes and saliva, especially among ixodids [28, 44]. A recently constructed database (TickSialoFam) has compiled and classified all these tick salivary protein sequences [45].

Regarding *O. erraticus*, neither its genome nor its sialotranscriptome have been sequenced and only six *O. erraticus* salivary proteins have been reported from an earlier proteomics study based on two-dimensional (2D) SDS-PAGE [46]. Therefore, the identity of the salivary bioactive proteins secreted by *O. erraticus* to the tick-host feeding interface remains unknown.

Because of their fast-feeding strategy, in argasid ticks, including *O. erraticus,* it is likely that the majority of the salivary molecules needed to complete blood-feeding are made prior to feeding and stored in their salivary glands before the ticks access the host. Once fed, *O. erraticus* ticks must synthesise and replace the bioactive proteins consumed during blood ingestion in order to be able to repeat a new trophogonic cycle. In this context, it can be assumed that (i) the salivary genes differentially upregulated between two consecutive feeding events are those that encode the bioactive proteins necessary to complete blood ingestion, and therefore, (ii) these proteins are potential antigen targets for immune or drug interventions aimed at preventing *O. erraticus* infestations and tick-borne pathogen transmission.

Accordingly, to determine both the identity of these proteins in *O. erraticus* and the time point after feeding when they are expressed, in the current study we aimed to (i) *de novo* assemble and annotate the sialotranscriptome of *O. erraticus*, (ii) evaluate the salivary gene expression dynamics along the trophogonic cycle and (iii) characterise the genes differentially upregulated after feeding. Among the latter, some particular groups of genes that were abundantly expressed and are functionally related to host attachment, blood ingestion and modulation of host defensive responses are discussed more thoroughly concerning selection of vaccine candidate antigens. This study sets the basis for future studies to understand the physiology of feeding in *O. erraticus* and to find effective antigen targets for development of anti-tick vaccines.

## Materials and methods

### Ticks and salivary gland collection

The *O. erraticus* ticks were obtained from the laboratory colony of IRNASA-CSIC (Salamanca, Spain), which was initiated from specimens captured in nature in the Salamanca Province (Spain) in the 1980s. The colony is kept at 28 °C, 85% relative humidity, 12-h light/12-h dark photoperiod and regularly fed on New Zealand white rabbits. All protocols involving tick feeding and rabbit handling were approved by the Ethical and Animal Welfare Committee of the IRNASA-CSIC and met the corresponding EU legislation (Directive 2010/63/EU).

Salivary glands were obtained from newly moulted, 3-month-old female ticks in three distinct physiological states along their trophogonic cycle: unfed (basal condition) and 7 and 14 days after feeding. Tick dissection and salivary gland removal were carried out in cold (4 °C) phosphate-buffered saline (PBS) at pH 7.4 treated with 1% diethyl pyrocarbonate (DEPC) and the salivary gland tissue was immediately stabilised in RNA later (Ambion, Austin, TX, USA). For each physiological state, two replicate samples of 50 pairs of salivary gland per sample were collected.

### RNA extraction, library construction and sequencing

Library preparation and sequencing were carried out at the Genomics Services of the Fundación Parque Científico de Madrid (Spain) (https://fpcm.es/en/servicios-cientificos/).

The six salivary gland samples (two biological replicates × three physiological states) were processed as described in the study by Oleaga et al. [39]. Briefly, salivary tissue was mechanically disrupted in the TissueLyser II (Qiagen, Valencia, CA, USA), and total RNA was extracted using the Monarch Total RNA Miniprep Kit" (New England BioLabs, Ipswich, MA, USA) according to the manufacturer's instructions and including on-column treatment with Turbo DNAse-free (Ambion) to remove any traces of contaminant DNA. RNA quality and concentration were assessed in the 2100 Bioanalyzer (Agilent, Santa Clara, CA, USA), showing RNA integrity number (RIN) values between 8.10 and 9.70 for the six samples.

One microgram of total RNA from each sample was used as input for library preparation with the NEBNext Ultra II Directional RNA Library Prep Kit for Illumina (New England BioLabs), following manufacturer recommendations for the Poly(A) mRNA protocol. Fragmentation time was reduced to 10 min in order to recover larger size fragments, in an attempt to facilitate assembly of pair-end reads.

The resulting libraries were validated and quantified in the 2100 Bioanalyzer (Agilent). An equimolecular library pool was made, purified using AMPure XP beads (Beckman Coulter, Brea, CA, USA) and titrated by quantitative PCR using the Kapa-SYBR FAST qPCR kit forLightCycler480 and a reference standard for quantification. The library pool was denatured and seeded on a NextSeq v2.5 flowcell (Illumina, San Diego, CA, USA) where clusters were formed and sequenced using a NextSeq 500 High Output kit v2.5 (Illumina) in a 2 × 150 pair-end read sequencing run on a NextSeq 500 sequencer (Illumina).

### Pre-processing and *de novo* assembly of the transcriptome

For every sample, raw reads were converted to FastQ format and subjected to quality control using FastQC (http://www.bioinformatics.babraham.ac.uk/projects/fastqc/). Read quality nucleotides were assessed using a PHRED quality score > 30 as a threshold. Fastq files > 100 nucleotides and having < 5% sequence indetermination were filtered and trimmed of adaptors and low-quality data (first 10 nucleotides) using Prinseq [47].

The transcriptome of each sample was *de novo* assembled using Oases [48], applying a k-mer range of 57–67. A merged transcriptome for each physiological state, as well as a consensus transcriptome from all six samples, was obtained using Minimus2 from the Amos package [49]. Redundant transcripts above a similarity threshold of 95% were eliminated using CD-HIT [50].

### Transcriptome annotation and characterisation

Coding sequences of the consensus transcriptome were searched using ORFPredictor software [51] and SeqEditor [52]. Only full open reading frames (ORFs) of 240 base pairs or longer (from ATG to stop codon) were selected for annotation.

Annotation was performed using the BLASTx and BLASTn programmes of the NCBI BLAST package with an *e*-value < 10^–05^ as the cut-off threshold against three databases: the NCBI non-redundant sequence database (NR) restricted to arthropoda [53, 54], Swiss-Prot [55] and the genome of *Ixodes scapularis* retrieved from Ensembl [56]. For this, the sequences selected in these databases (21 November 2019) were downloaded and combined in a custom database containing 8,048,569 sequences.

The predicted polypeptides were characterised as follows: (i) functional characterisation including identification of conserved protein domains and protein families according to the Pfam terms [57] included in the Uniprot and Interpro databases, respectively [58]; assignation of Gene Ontology (GO) terms [59] based on Uniprot accessions regarding biological process, molecular function and cellular component categories using Worksheet tool of the GPRO Suite; and metabolic pathways analysis from Kyoto Encyclopedia of Genes and Genomes (KEGG) [60] using the enzyme codes (EC) associated to functional GO terms as queries; (ii) topological characterisation, including detection of transmembrane domains, glycosil-phosphatidyl-inositol (GPI) anchors and signal peptide using, respectively, the following tools: TMHMM [61], PredGPI [62] and signalP-5.0 [63]; (iii) antigenicity prediction with Vaxijen 2.0 [64] using 0.5 as a threshold cut-off. Topological characterisation and antigenicity prediction were carried out to annotated transcripts only.

The completeness of the *O. erraticus* salivary gland transcriptome was evaluated using BUSCO (Benchmarking Universal Single-Copy Orthologs) by comparing the transcriptome against a set of highly conserved single-copy orthologs of the known ancestral Arachnida proteins (arachnida_odb10, creation date: 2020-08-05, number of species: 10, number of BUSCOs: 2934) [65].

### Differential expression analysis

To perform differential expression and enrichment analyses between the distinct conditions, raw sequence reads from each library were mapped against the consensus transcriptome using the mapper Bowtie2 [66]. The average alignment rate was higher than 97.4% for all libraries; thus, confirming the quality of the consensus transcriptome. Corset [67] was applied to hierarchically cluster short transcripts into long genes for downstream analyses, resulting in a cluster file grouping the 103,041 consensus transcripts into 97,343 transcript clusters and a count file summarising the read counts obtained per cluster from each of the 6 paired-end libraries mapped to the consensus transcriptome. Read counts per transcript were used as input to EdgeR package [68] to perform three differential expression analyses between physiological states: 7-day fed *vs* unfed (7 *vs* 0), 14-day fed *vs* 7-day fed (14 *vs* 7) and 14-day fed *vs* unfed (14 *vs* 0). Transcripts showing a log_2_ fold-change (log_2_ FC) ≥ 1 or ≤ – 1 and an adjusted *p*-value < 0.05 after False Discovery Rate (FDR) correction applied by EdgeR using the Benjamini-Hochberg method [69] were considered differentially expressed.

### Gene ontology (GO) and metabolic pathway enrichment analyses

GO and metabolic pathway enrichment analyses of the differentially expressed transcripts were performed using the GOseq package [70]. Metabolic pathway enrichment analysis of the differentially expressed transcripts was performed using the KEGG database. For this, the enzyme codes (EC) associated to the enriched GO terms were used to download KEGG maps [71] and recover information of the pathways involved, which were annotated using the GPRO suite [72]. Enriched GO terms and metabolic pathways showing adjusted *p*-values < 0.05 (FDR correction) in the resulting Wallenius distribution were considered significant.

### Data availability

The transcriptome data generated for this study were deposited at the National Institute for Biotechnology Information (NCBI) under bioProject ID PRJNA666995 and biosample accessions SAMN16339901, SAMN16339902, SAMN16339903, SAMN16339904, SAMN16339905, SAMN16339906. This Transcriptome Shotgun Assembly project has been deposited at DDBJ/EMBL/GenBank under the accession GIXX00000000. The version described in this paper is the first version, GIXX01000000.

### Pipelines

The protocols and tools for pre-processing, *de novo* assembly, annotation and differential expression analyses were executed using the DeNovoSeq and the RNAseq tools of the GPRO suite [72].

## Results and discussion

### Sequencing and *de novo* assembly of the *O. erraticus* sialotranscriptome at three physiological conditions

In this study we aimed to *de novo* assemble and annotate the sialotranscriptome of *O. erraticus* females as well as assess the salivary gene expression dynamics along its trophogonic cycle. For this, we prepared replicated transcriptome samples from the salivary glands of females taken in three different physiological conditions: unfed females (OE0_1, OE0_2) and fed females at 7 (OE7_1, OE7_2) and 14 (OE14_1, OE14_2) days after blood-feeding.

The six samples were sequenced using illumina RNAseq paired-end technology and the resultant FastQ libraries were subjected to quality control and *de novo* assembly, obtaining 82,838 and 88,590 transcript contigs for replicated samples from unfed females (0 days), 61,373 and 53,779 for samples from 7-day fed females, and 73,236 and 77,721 for samples from 14-day fed females (Additional file 1: Table S1). For the assembly of the primary transcripts only reads > 100 nucleotides were used.

Consensus transcriptomes for each physiological state resulted in 106,223, 75,491 and 93,846 transcript contigs for unfed, 7-day fed and 14-day fed, respectively. Additionally, a consensus transcriptome was obtained merging the six assemblies constructed *de novo*. This consensus transcriptome consisted of 103,041 transcripts with the following metrics: N50 transcript length 1884 base pairs (bp), mean transcript size 1194 bp and longest transcript size 26,653 bp (Additional file 1: Table S1; Table 1).

**Table 1:** *O. erraticus* salivary gland transcriptome assembly and annotation statistics

Summary	0 days	7 days	14 days	Merged
Transcriptome assembly statistics				
Total transcriptome size	130,120,686	93,400,441	118,099,223	123,061,068
Number of transcripts	106,223	75,491	93,846	103,041
Longest transcript (bp)	17,265	17,479	26,653	26,653
Shortest transcript (bp)	100	100	100	100
% Transcripts > 1 kb	41.32%	40.83%	41.52%	39.97%
Mean transcript size (bp)	1224	1237	1258	1194
Median transcript size (bp)	815	797	817	780
N50 transcript length (bp)	1875	1979	1978	1884
L50 transcript count	19,869	13,592	16,813	18,414
Transcriptome annotation statistics				
Transcript clusters				97,343
Transcripts > 1 RPKM				28,061
Full ORF > 240 bp				22,007
Annotated in at least one database				18,961 (86.0%)
Assigned with EC numbers				3608 (16.4%)
Assigned with GO terms				9249 (42.0%)
Assigned to KEGG pathways				3725 (16.9%)
Assigned with PFAM identifiers				6619 (30.0%)
Assigned with InterPro identifiers				9116 (41.7%)
Number of non-redundant predicted proteins				9355

Samples were taken from female ticks before feeding (0 days) and 7 and 14 days after feeding

Redundancy filter of these 103,041 transcripts resulted in 97,343 transcript clusters, of which 28,061 had an expression level > 1 read per kilobase per million reads (RPKM), which represented 28.82% of the sequences, but 94.25% of the total expression measured in RPKM. We used 1 RPKM as the expression level threshold to exclude lowly expressed transcripts, which most likely represented background expression or assembly artefacts [73]. Accordingly, nearly 75,000 transcripts were excluded, which comprised < 6% of the total expression.

Among these 28,061 transcripts, we selected those containing a predicted full ORF > 240 bp from the start (ATG) to the stop codon. This resulted in a final transcriptome of 22,007 high-confidence transcripts, which were subjected to functional annotation, characterisation, and differential expression and enrichment analyses (Table 1).

Completeness of the final transcriptome, as evaluated using BUSCO, revealed that 84.2% of conserved genes across Arachnida were present (2469 out of 2934). Complete and single-copy genes found were 47.0% (*n* = 1379), complete and duplicated genes found were 37.2% (*n* = 1090), fragmented genes found were 1% (*n* = 30), and missing were 14.8% (*n* = 435).

### Functional annotation and classification according to GO terms, protein domain families and biological pathways

Blast searching of the 22,007 transcripts against the NCBI NR, Swissprot and *Ixodes scapularis* genome databases delivered 18,961 (86.16%) annotated sequences with an e**-**value < 10^–05^, of which 14,219 (75.0%) showed a sequence similarity > 60% (Additional file 2: Table 2). The remaining 3046 sequences (13.84%) did not show significant homology to any sequence in the referred databases. These sequences may represent still-unknown proteins, misassembled coding sequences of no biological significance or even long non-coding RNAs, which can be difficult to distinguish from misassembled ORFs [73, 74]. For the 18,961 annotated ORFs, a total number of 9355 non-redundant predicted proteins with unique accession codes were found (Table 1).

**Table 2:** Metabolic pathways differentially enriched in the salivary glands of *O. erraticus* in each comparison

Class	Pathway ID	Pathway	Seq in pathway	Num DE seq in pathway		
				7 *vs* 0	14 *vs* 7	14 *vs* 0
Lipid metabolism	map00590	Arachidonic acid metabolism	274	181	61	148
	map00564	Glycerophospholipid metabolism	279	176	61	144
	map00565	Ether lipid metabolism	242	176	61	144
	map00592	Alpha-linolenic acid metabolism	235	176	61	144
Carbohydrate metabolism	map00630	Glyoxylate and dicarboxylate metabolism	52	–	–	17
Amino acid metabolism	map00250	Alanine, aspartate and glutamate metabolism	40	–	–	11
	map00350	Tyrosine metabolism	34	–	–	9
Energy metabolism	map00910	Nitrogen metabolism	17	9		8
Xenobiotics biodegradation and metabolism	map00983	Drug metabolism-other enzymes	40	15		–

7-day fed *vs* unfed (7 *vs* 0), 14-day fed *vs* 7-day fed (14 *vs* 7) and 14-day fed *vs* unfed (14 *vs* 0). Seqs in pathway: number of sequences included in the pathway. Num DE seq in pathway: number of enriched sequences in the pathway

The 18,961 annotated transcripts were functionally characterised using the Gene Ontology (GO), Protein Domain Families (Pfam) and Biological Pathways (KEGG) databases, associated to Swissprot annotations.

**Fig. 1:**
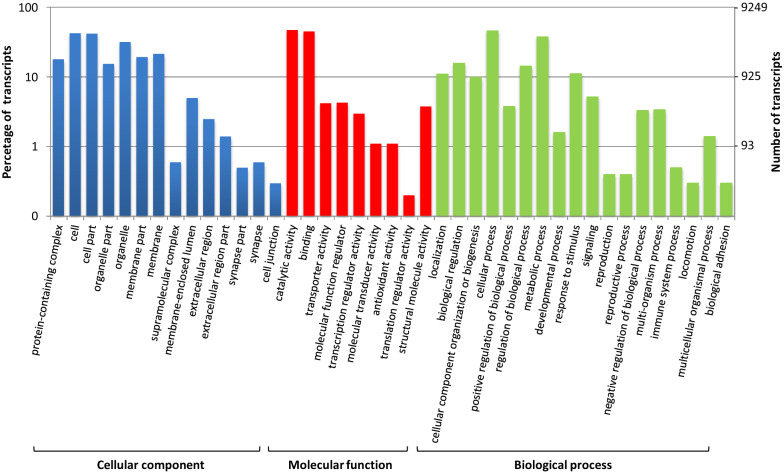
Gene ontology (GO) distribution of *O. erraticus* salivary gland transcriptome. Level 2 GO terms of cellular components (blue), molecular functions (red) and biological processes (green) were visualized using WEGO (Web Gene Ontology Annotation Plot). These included 18,719 cellular components, 10,222 molecular functions and 15,476 biological processes. Bars represent the percentage and number of annotated transcripts in each category

Up to 9249 transcripts were assigned GO terms (Additional file 2: Table S2). These included 18,719 cellular components, 10,222 molecular functions and 15,476 biological processes, which were visualised using the Web Gene Ontology Annotation Plot (WEGO) [75]. Figure 1 represents the transcripts classified according to cellular component, molecular function and biological process, using level 2 GO terms. The cellular components were classified into 14 categories, of which the 7 more abundantly represented were cell (*n* = 3969), cell part (*n* = 3907), organelle (*n* = 2939), membrane (*n* = 1994), membrane part (*n* = 1795), protein-containing complex (*n* = 1667) and organelle part (*n* = 1435). Classification by molecular function resulted in nine categories. The more abundantly represented had by far catalytic (*n* = 4412) and binding activity (*n* = 4187); the remaining categories were significantly less represented and included molecular function regulator (*n* = 397), transporter activity (*n* = 386), structural molecule (*n* = 349), transcription regulation (*n* = 274), antioxidant activity (*n* = 101), molecular transducer (*n* = 98) and translation regulation (*n* = 18). Classification of biological processes resulted in 18 categories. The eight more abundant were: cellular processes (*n* = 4281), metabolic process (*n* = 3518), biological regulation (*n* = 1,458), regulation of biological process (*n* = 1335), response to stimulus (*n* = 1037), localisation (*n* = 1019), cellular component organisation or biogenesis (*n* = 916) and signalling (*n* = 477). This GO distribution is similar to that reported for the mialomes of *O. erraticus* and *O. moubata* females [31, 32] and for the more recently reported sialotranscriptome of *O. moubata* females [43].

**Fig. 2:**
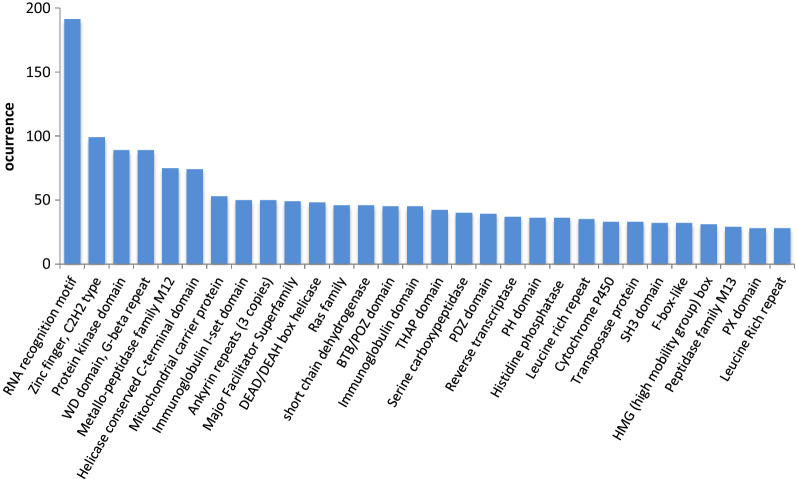
Top 30 Pfam domain occurrences in the *O. erraticus* predicted proteins. A total of 9828 Pfam domains were observed in the *O. erraticus* proteins, of which 2,458 were unique. Up to 6619 of the proteins contained at least one Pfam domain

Analysis of the sialotranscriptome in the Pfam database assigned up to 9,828 Pfam domains (of which 2458 were unique) to 6619 transcripts (Additional file 3: Table S3). The top 30 assigned Pfam domains are represented in Fig. 2. The more frequently assigned were the RNA recognition motif (a.k.a. RRM, RBD, or RNP domain) (*n* = 191), zinc finger, C2H2 type (*n* = 99), protein kinase domain (*n* = 89), WD domain, G-beta repeat (*n* = 89), metallo-peptidase family M12 (*n* = 75) and helicase conserved C-terminal domain (*n* = 74). These protein domains are mostly the same as those more frequently found in the sialotranscriptome of *O. moubata* [43], which were also abundantly represented in the mialomes of *O. erraticus* and *O. moubata* [31, 32]. Indeed, these domains are highly abundant in eukaryotic cells where they participate in a wide range of biological functions such as apoptosis, cell cycle control, chromatin remodelling, cytoskeletal organisation, development, intracellular transport, ribosome biogenesis, transcriptional regulation, signal transduction and immune responses [76–79].

**Fig. 3:**
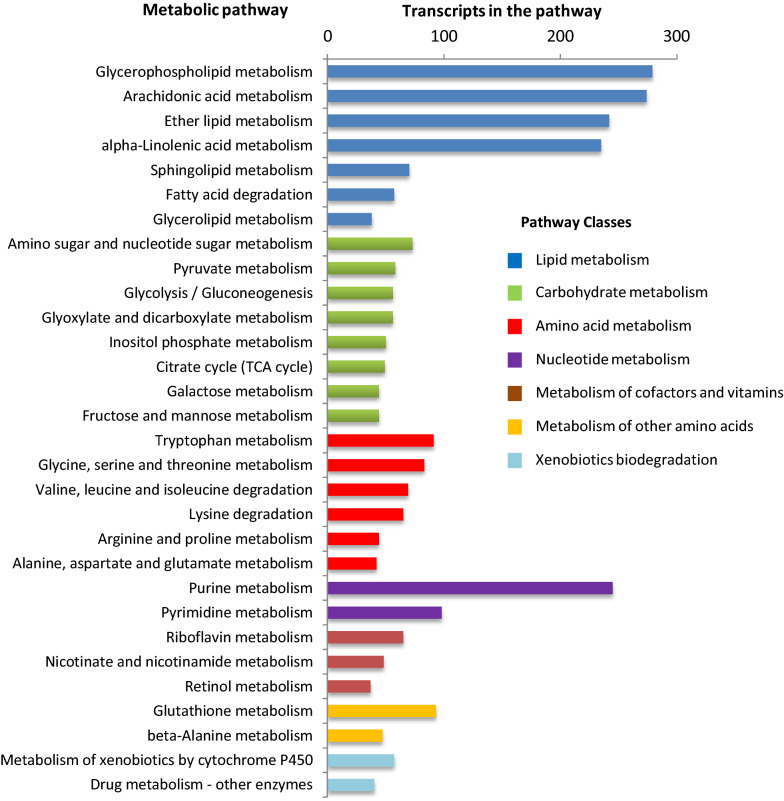
The top 30 most abundant KEGG biological pathways identified in the sialotranscriptome of *O. erraticus* include 2687 transcripts grouped in 7 pathway classes

Analysis of the annotated transcripts in the KEGG database allowed the identification of active biological pathways in the salivary glands. In this way, 3725 *O. erraticus* salivary sequences were assigned to 627 enzymes, 100 pathways and 13 pathway classes (Additional file 4: Table S4). The top 30 most represented pathways classified into seven pathway classes and included 2687 (72.12%) of the 3725 enzyme sequences (Fig. 3). These enzymes mostly belong to pathways involved in lipid (1180 sequences), carbohydrate (416), amino acid (382 sequences) and nucleotide (335 sequences) metabolism.

### Differential expression of the sialotranscriptome

We sought to identify the *O. erraticus* salivary genes that were differentially expressed as a function of time after blood-feeding.

As with other fast-feeding argasid ticks, it is assumed that the bioactive components of the saliva of *O. erraticus* are previously synthesised, stored in its salivary glands and ready to be secreted after accessing the host. Thus, only basal gene expression can be expected before feeding. After completing their blood meal, *O. erraticus* ticks must synthesise and replace the pool of bioactive molecules that they have consumed during feeding in order to be able to feed again. To determine which are bioactive proteins and when are they expressed, we investigated the salivary gene expression dynamics at three points of time along the trophogonic cycle: before feeding (basal condition) and at 7 and 14 days after blood-feeding. These sampling points were selected as intermediate points based on the duration of the trophogonic cycle of *O. erraticus* female ticks, which typically takes 19–21 days at 28 °C from feeding to oviposition [80].

**Fig. 4:**
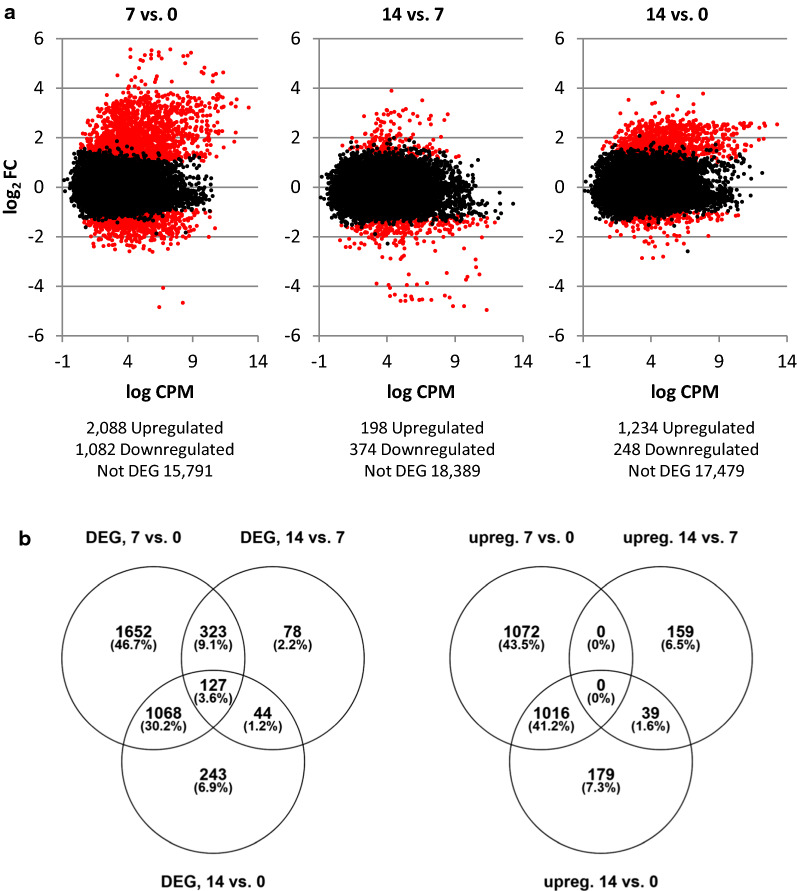
Differential expression patterns of the *O. erraticus* sialotranscriptome. **a** Volcano plots representing the log_2_ fold change (log_2_FC) against the log counts per million (log CPM) for every transcript across the three comparisons. Transcripts with FDR < 0.05 and log_2_FC ≥ 1 or ≤ -1 were considered differentially expressed and are plotted in red. Transcripts outside this range were not differentially expressed (not DEG) and are plotted in black. **b** Venn diagrams showing the number of differentially expressed genes (DEG, left panel) and of differentially upregulated genes (upreg., right panel) in each comparison. Comparisons: 7 *vs* 0, 7-day fed *vs* unfed; 14 *vs* 7, 14-day fed *vs* 7-day fed and 14 *vs* 0, 14-day fed *vs* unfed

Differential gene expression in *O. erraticus* salivary glands was then evaluated by comparing the gene expression levels among these three physiological states: 7-day fed *vs* unfed (7 *vs* 0), 14-day fed *vs* 7-day fed (14 *vs* 7) and 14-day fed *vs* unfed (14 *vs* 0) (Additional file 5: Table S5, Fig. 4).

The highest differential expression was observed at 7 days after feeding (7 *vs* 0) with 3170 differentially expressed transcripts, of which 2088 were upregulated (log_2_FC > 1, and FDR < 0.05) and 1082 were downregulated (log_2_FC < − 1, and FDR < 0.05) (Fig. 4a). Between 7 and 14 days after feeding (14 *vs* 7), there were only slight variations, as only 122 additional differentially expressed transcripts were detected (Fig. 4b). Comparison between the basal condition and 14 days after feeding (14 *vs* 0) showed 1482 differentially expressed transcripts, of which 1234 were upregulated. Remarkably, the 82.3% of the transcripts that were differentially upregulated at 14 days after feeding (1016) had already been differentially upregulated at 7 days after feeding (Additional file 5: Table S5, Fig. 4b). These results indicate that most of the differentially upregulated gene expression in salivary glands occurred in the first 7 days after feeding, being less important from day 7 after feeding onwards.

As a whole, these data suggest that the bioactive salivary proteins secreted into the host during blood ingestion are mainly synthesised and replaced in the first 7 days after blood meal ingestion and encourage further analyses of salivary gene transcription in shorter time intervals, as, for example, every 24 h post-detachment to 7 days. This will provide more precise information on gene transcription regulation for each salivary protein family and will show whether the different protein families and the different members inside a particular family are differentially expressed over time, as has been observed in ixodid ticks during feeding. In ixodids, which are slow-feeding ticks and synthesise part of their saliva components during the feeding process, salivary gene expression is temporally regulated along feeding resulting in several consecutive changes in the composition of their sialome and saliva. This process is known as “sialome and saliva switching” and is considered a mechanism to evade the host immune response and adapt to feeding on different host species [45, 73, 81–83].

In argasid ticks, sialome and saliva switching has never been described. This is not surprising owing to the short duration of feeding in argasids, which would make sialome and saliva switching an unnecessary evasion mechanism during the feeding process.

However, it should be mentioned that in argasids the gene expression pattern during blood-feeding has never been studied and it remains unknown despite the fact that it may be an important issue. Regarding *O. erraticus,* this analysis is technically complex as most females complete feeding in approximately 15 min. This technical complexity may explain the lack of studies on salivary gene expression during feeding in argasids. Furthermore, only a handful of argasid sialomes have been published hitherto and studies on argasid salivary gene expression patterns before and after feeding have just started.

### Enrichment of gene ontologies and metabolic pathways

**Fig. 5:**
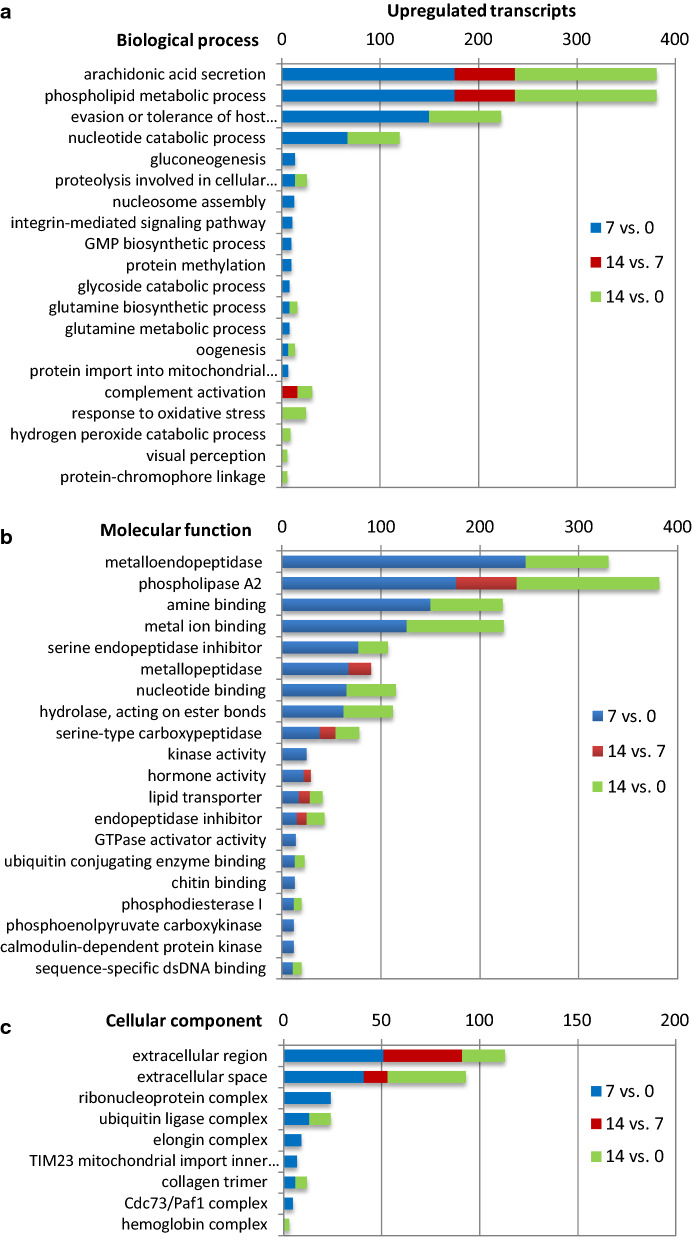
Enrichment of gene ontology (GO) terms in the *O. erraticus* sialotranscriptome after feeding. Top 20 significantly overrepresented GO terms of biological process (**a**), molecular function (**b**) and cellular component (**c**) showing the number differentially expressed genes between 7-day fed *vs* unfed females (7 *vs* 0), 14-day fed *vs* 7-day fed females (14 *vs* 7) and 14-day fed *vs* unfed females (14 *vs* 0)

The results of the GO enrichment analyses are offered in Additional file 6: Table S6, which compiles the significantly overrepresented GO terms assigned to the differentially expressed salivary genes. Additionally, Fig. 5 shows the significantly overrepresented top 20 biological processes, molecular functions and cellular components in the three comparisons.

Up to 98 GO terms were significantly overrepresented (FDR < 0.05) in at least one of the comparisons: 36 biological processes, 53 molecular functions and 9 cellular components. For the biological process, enrichment analysis showed that 20, 11 and 20 GO terms were significantly overrepresented in comparisons 7 *vs* 0, 14 *vs* 7 and 14 *vs* 0, respectively (Additional file 6: Table S6). Figure 5a shows that among the overrepresented top 20 biological processes, the categories with the highest numbers of upregulated sequences corresponded to proteins involved in arachidonic acid secretion, phospholipid metabolic processes, evasion or tolerance of host defence response and nucleotide catabolic processes. For molecular function, enrichment analysis revealed that 37, 10 and 34 GO terms were significantly overrepresented in comparisons 7 *vs* 0, 14 *vs* 7 and 14 *vs* 0, respectively (Additional file 6: Table S6). Figure 5b shows that, at 7 and 14 days after feeding, the molecular functions assigned to the highest number of sequences were metalloendopeptidase activity, phospholipase A2 (PLA2) activity, amine-binding, metal ion-binding and serine-type endopeptidase inhibitor activity. All these ontologies are related to functional groups and protein families highly upregulated at 7 and 14 days after feeding. These included proteins with PLA2 activity, 5′-nucleotidases/apyrases, lipocalins, metallopeptidases and protease inhibitors, all with important functions in the blood-feeding process [84]. Regarding cellular compartments, only eight, two and five GO terms were significantly overrepresented in comparisons 7 *vs* 0, 14 *vs* 7 and 14 *vs* 0, respectively (Additional file 6: Table S6). The top overrepresented GO terms correspond to sequences assigned to extracellular compartments, most likely related to the synthesis and secretion of proteins in saliva (Fig. 5c).

**Fig. 6:**
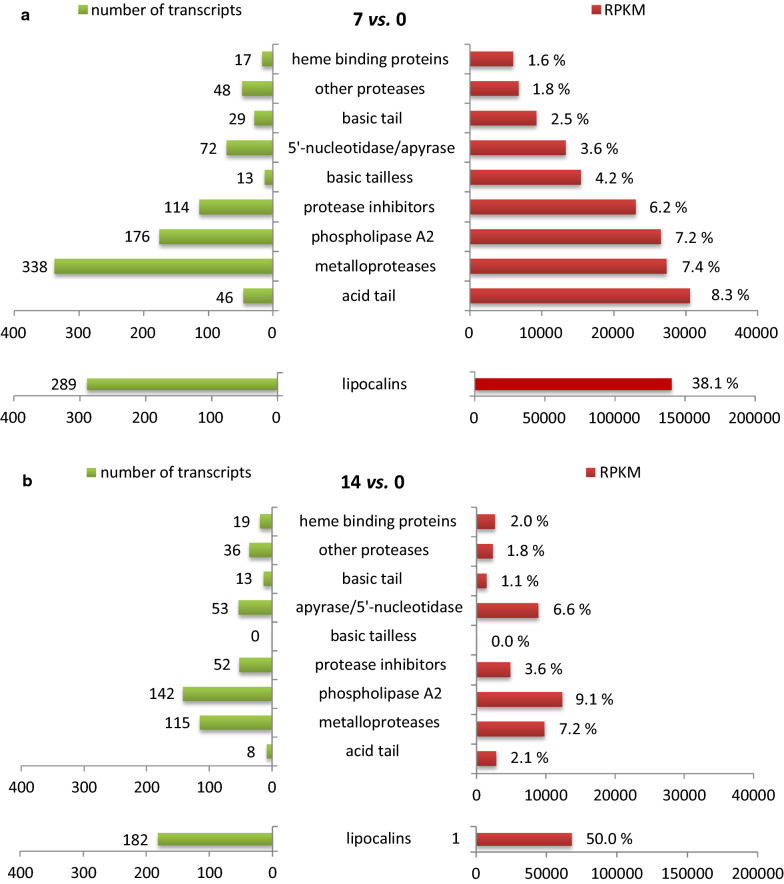
Number of upregulated annotated transcripts (green bars) and expression level in RPKM (red bars) for each protein group/family in comparisons between 7-day fed *vs* unfed females (7 *vs* 0) (**a**) and 14-day fed *vs* unfed females (14 *vs* 0) (**b**). Percentages at the red bar ends represent the ratio between the expression level of each group/family and the total expression in RPKM of the whole annotated upregulated transcriptome

The analysis of the enriched metabolic pathways in the differentially expressed genes revealed (i) significant overrepresentation (FDR < 0.05) of nine biological pathways and five pathway classes in at least one of the three comparisons and (ii) significant overrepresentation of six, four and eight biological pathways in comparisons 7 *vs* 0, 14 *vs* 7 and 14 *vs* 0, respectively (Additional file 7: Table S7; Table 2). This pattern of enrichment along the trophogonic cycle is parallel to the patterns observed for GO enrichment and differential gene upregulation as most pathways are enriched in comparisons 7 *vs* 0 and/or 14 *vs* 0, accruing 733 and 625 sequences, respectively. Three pathways in the classes “carbohydrate metabolism” and “amino acid metabolism” were enriched in comparison 14 *vs* 0 only (37 sequences) while the sole pathway in the class “xenobiotics biodegradation and metabolism” was enriched in comparison 7 *vs* 0 only (15 sequences). As a whole, the overrepresented pathways are related to the metabolism of lipids, amino acids, carbohydrates, energy and xenobiotics, with lipid metabolism pathways largely containing the highest number of overexpressed sequences (709 and 580 for comparisons 7 *vs* 0 and 14 *vs* 0, respectively) (Table 2). This observation is in agreement with the high number and expression level of upregulated transcripts annotated as enzymes with PLA2 activity (Fig. 6), which participate in several lipid metabolic pathways, such as the glycerophospholipid, arachidonic acid, ether lipid and alpha-linolenic acid metabolism (Fig. 3).

### Main protein groups and families upregulated after feeding

We were interested in the protein groups and families that participate in tick attachment and feeding, including those involved in the modulation of host defensive responses, because these proteins may be suitable targets for development of vaccines for the prevention and control of tick infestations and tick-borne diseases. In this setting, we assumed that the salivary genes differentially upregulated after feeding would indeed be those encoding the bioactive proteins that *O. erraticus* ticks need to be able to feed again.

Therefore, among the protein groups and families that were upregulated at 7 and 14 days after feeding (comparisons 7 *vs* 0 and 14 *vs* 0), we focused on those showing the highest expression levels in RPKM. These were lipocalins, metalloproteases, proteases other than metallo, protease inhibitors, proteins with PLA2 activity, 5′-nucleotidases/apyrases, acid tail proteins, basic tail proteins, basic tailless proteins and heme-binding proteins (Fig. 6 and Tables 3, 4, 5, 6). Together, these groups accrue 80.9% and 83.5% of expression of the whole annotated upregulated transcriptome at 7 and 14 days after feeding, respectively. Not surprisingly, most of these protein groups and families are also the most abundantly represented in the sialomes of other argasid and ixodid tick species [41, 43, 45].

**Table 3:** Lipocalins and 5′-nucleotidases/apyrases differentially upregulated 7 days after feeding (7 *vs* 0)

Description	Accession	No. transcripts	RPKM 0	RPKM 7	Log_2_FC
Lipocalins					
Lipocalin	ABI52664, ABI52661, ABI52807, ASV59774	61	2930.76	13,014.59	1.10–3.98
Monotonin	ABI52654	4	569.23	5714.97	3.11–3.52
Moubatin, moubatin-like	ABR23415, ABR23399, ABR23347, ABR23414, ABR23462, ABR23457, ABR23458, ABR23380, ACB70378	128	6039.08	52,417.05	1.06–4.34
Salivary lipocalin	ACB70384, ABR23445, ACB70352, ABR23418, ACB70348, ABR23386, ABR23385, ACB70377, ABR23357, ACB70386, ABR23394	138	9266.83	63,248.20	1.12–3.81
Salivary secreted lipocalin	ABR23443, ACB70358	9	1104.44	4,980.50	1.43–3.39
Savicalin	ADI60053	2	176.64	837.51	2.13–2.25
TSGP2	AAN76829	2	38.49	111.91	1.51–1.56
5′-Nucleotidases/apyrases					
5′-Nucleotidase, putative	B7QDH9, B7QKF1	3	69.35	155.018	1.15–1.30
5′-Nucleotidase, ecto, putative	B7QI36	2	16.14	37.64	1.19–1.36
5′-Nucleotidase/putative apyrase	ABS30896, ABS30897, ACB70391	42	1239.92	4759.81	1.21–2.64
5′-Nucleotidase-like	XP_027236452	2	52.08	136.51	1.35–1.40
Apyrase	AGJ90350, ETN57989	23	2050.19	8,280.60	1.15–2.32

For each protein description, the accession codes and number of transcripts assigned, the average expression level in RPKM for unfed (RPKM 0) and 7 days fed (RPMK 7) females, and the range of log_2_FC reached are provided

For each of these groups/families, Fig. 6 shows the number of upregulated transcripts, their expression level in RPKM, and their expression ratio (in percentage) with respect to the total expression in RPKM of the whole annotated upregulated transcriptome at 7 (Fig. 6a) and 14 (Fig. 6b) days after feeding.

At 7 days after feeding, lipocalins are the family that shows the highest expression level (140,291.67 RPKM) and the second higher number of upregulated transcripts (*n* = 289). Lipocalins represent up to 38.1% of total expression in the annotated upregulated transcriptome, which is more than five-fold the expression level of the remaining groups/families (Fig. 6a). In these other groups, their expression ratio ranged between 8.3–1.6% for acid tail proteins, metalloproteases, PLA2s, protease inhibitors, basic tailless proteins, 5′-nucleotidases/apyrases, basic tail proteins, non-metalloproteases and heme-binding proteins. Among these groups, metalloproteases and PLA2s showed the highest number of transcripts (338 and 176, respectively), whereas acid tail proteins showed a relatively low number of transcripts (*n* = 46) in relation to their high expression ratio (8.3%). A similar situation was observed in the *O. rostratus* sialotranscriptome, where only seven contigs annotated as acid tail proteins accounted for up to 21% of all RNA expressed in salivary glands [79].

At 14 days after feeding the expression pattern of most groups showed sharp reductions in both the transcript number and expression level; this was particularly pronounced for acid tail, basic tail and basic tailless proteins. By contrast, heme-binding proteins showed slight increases in their upregulated transcript numbers and expression ratios, although not in their expression levels, which remained below the level reached at 7 days post-feeding (Fig. 6b).

These results show that these particular groups and families of bioactive proteins are mainly upregulated in the first 7 days after feeding, their upregulation beyond this time point being less important, and confirm that important bioactive salivary proteins secreted into the host during blood ingestion are synthesised and replaced in the first 7 days after a blood meal.

### An insight into the most abundantly overrepresented proteins at 7 days after feeding

Due to their potential value as antigen targets of the aforementioned protein groups and families, in this section the most abundantly overrepresented proteins are discussed in more depth.

#### Lipocalins

The lipocalin family is one of the largest, most diverse and abundant in the sialomes of ticks. These secreted proteins play important functions in evading the host haemostatic, inflammatory and innate immune responses, mainly as kratagonists of biogenic amines and eicosanoids [29, 41, 43, 45, 46, 85, 86].

In this study, we found 289 lipocalin transcripts (Table 3; Fig. 6a). Up to 145 of them (50%) were annotated with the InterPro code “IPR002970 Tick histamine-binding protein” (Additional file 2: Table S2), which strongly suggests that for *O. erraticus* to be able to feed, it is crucial to avoid the inflammatory reaction induced by the release of histamine at the feeding lesion. Among the remaining transcripts, up to 130 were annotated as moubatin, moubatin-like or TSGP2. All of them belong to the moubatin clade of lipocalins, which include proteins that inhibit platelet and neutrophil aggregation by scavenging thromboxane A2 (TXA2) and leukotriene B4 (LTB4), respectively, and proteins that inhibit complement activation by sequestering the C5 component [39, 87–89]. The abundant representation of moubatins in the upregulated sialotranscriptome underlines that blocking the host haemostasis and innate immunity is also critical for *O. erraticus* to feed. Two additional transcripts annotated as savicalin were also identified in this sialotranscriptome. Savicalin was found in the haemocytes, midgut and ovaries of *Ornithodoros kalaharensis,* formerly designated *O. savignyi* [90], but not in its salivary glands. Savicalin is upregulated in the midgut and ovaries after feeding and in haemocytes after bacterial challenge, suggesting its involvement in tick development after feeding and/or in anti-microbial defence [91]. Thus, the presence of savicalin in tick salivary glands/saliva is novel and its function here is unknown, but it might be related to protection against pathogens acquired during feeding.

The lipocalin expression pattern described here for *O. erraticus* is similar to that found for the salivary lipocalins of other soft ticks such as *O. rostratus* and *O. moubata* [41, 43].

#### 5'-Nucleotidases/apyrases

These enzymes hydrolyse ATP and ADP to AMP and are common in the saliva of hematophagous arthropods. They interfere with host haemostasis by hydrolysing the ADP released at the feeding site by the injured endothelial cells. This prevents platelet and neutrophil aggregation and thrombus formation, allowing blood flow to the bite site and thereby facilitating tick blood-feeding [92–94].

Up to 72 upregulated transcripts of 5′-nucleotidase/apyrase were found in the sialotranscriptome of *O. erraticus*, accounting for 3.6% of total expression at 7 days after feeding (Table 3; Fig. 6a). These enzymes have been abundantly found in the sialomes of hard and soft ticks, including *O. parkeri*, *O. coriaceus*, *O. rostratus*, *O. kalaharensis* and *O. moubata*, underscoring the important anti-haemostatic role of these enzymes in the tick-feeding process [37, 38, 41, 43, 44, 85, 92]. Indeed, blocking of the apyrase function by host immunisation with a recombinant apyrase protein significantly reduced feeding in *O. moubata* ticks, demonstrating that 5'-nucleotidases/apyrases are promising candidate antigens for anti-tick vaccine development [27].

#### Proteases

Up to 386 upregulated transcripts annotated as proteases were found in the current study, most of which were metalloproteases (338 transcripts), followed by serine proteases (47 transcripts) and cysteine proteases (one transcript) (Table 4; Fig. 6a).

**Table 4:** Proteases differentially upregulated 7 days after feeding (7 *vs* 0)

Description	Accession	No. transcripts	RPKM 0	RPKM 7	Log_2_FC
Metalloproteases					
A disintegrin and metalloproteinase with thrombospondin motifs 8 (ADAMs)	KFM57926	1	10.98	24.49	1.16
Carboxypeptidase Q	AMO02552	1	12.20	40.37	1.73
Endothelin converting enzyme	KZC14076, XP_022117928, B7QLL2, B7PLU3	6	79.37	205.27	1.04–1.93
Hypothetical protein B7P43_G04849	PNF39037	1	3.64	10.07	1.46
Hypothetical protein DAPPUDRAFT_187662	EFX85417	2	4.50	11.38	1.31–1.36
Hypothetical protein Phum_PHUM474680	EEB17615	2	2.97	12.91	2.11–2.14
Hypothetical protein TSAR_002326	OXU27510.1	1	6.08	25.88	2.09
Membrane metallo-endopeptidase	ODM96912, XP_015911874, KFM79078	3	18.65	66.46	1.58–2.23
Metalloprotease	BAE00066, ABI52776, BAF43575, ABI52714, ABI52747, ABI52815, ABI52719, BAE72661, ABI52779, ABI52738, BAE72664, ABI52662, ABI52652, B7Q2B8, B7PPE9, B7Q2C1, B7Q4I3, DAA34338	125	4024.53	14,474.84	1.08–3.24
Metalloprotease 1	AIE44747, AIE44750, AIE44753	4	103.52	277.06	1.04–1.59
Metalloprotease 2	AIE44748	1	26.01	57.17	1.14
Metalloprotease 4	AIE44755	7	127.35	290.99	1.07–1.61
Metis1 protein	CAO00625	14	204.99	574.06	1.09–2.25
Metis2 protein	CAO00626	4	21.61	61.89	1.32–1.61
Metis3 protein	CAO00627	32	494.29	1,754.92	1.00–2.41
Metis4 protein	CAO00628	9	335.26	1,281.06	1.08–2.61
Metis5 protein	CAO00629	1	14.89	86.24	2.53
M20 domain-containing peptidase	B7P153	3	4.84	11.43	1.14–1.24
Neprilysin	XP_001849326, ISCW024547-PA, B7QAF9, B7Q3V5, B7PL32, AAL91975, DAA34245	14	868.32	2,009.44	1.03–2.49
Neprilysin-1	XP_019696599, XP_022255642	9	88.88	340.64	1.12–2.55
Neprilysin-2	XP_015795185, XP_026818640, XP_008476829, XP_023343136, XP_019868485	9	294.8	845.97	1.17–2.06
Neprilysin-4	XP_024228249, XP_026469709	2	40.41	126.42	1.63–1.65
Neprilysin-11	RWS07493, XP_001600059	4	39.64	89.24	1.00–1.44
Peptidase M12B domain-containing protein	B7QM92	15	518.28	2,083.55	1.03–3.55
Peptidase-like protein	ADF28505	1	17.18	55.67	1.70
Salivary gland metalloprotease	AAZ39657, AAZ39658, AAZ39659 EEC19961, EEC18166, DAA34140, DAA34198	20	268.52	791.84	1.16–1.84
Secreted metalloprotease	B7Q2C0, B7QM91, AAM93653	7	77.82	335.34	1.39–2.98
Truncated metalloprotease	ABR23495	7	199.09	635.98	1.09–2.05
Venom metalloproteinase 3-like	XP_008547408, XP_014223136	2	21.04	75.98	1.85–1.86
Venom metalloproteinase antarease-like TtrivMP_A	XP_018494555, XP_022665601	3	35.09	128.99	1.30–1.90
Zinc-dependent metalloprotease	ACB70344	28	159.78	572.61	1.00–3.37
Serine proteases					
Carboxypeptidases	B7QF76	14	149.98	383.33	1.28–1.47
Lysosomal protective protein	XP_013784602	3	9.64	28.98	1.47–1.77
Retinoid-inducible serine carboxypeptidase	XP_015927829, KFM77940, XP_013787581	11	46.45	234.46	1.78–2.73
Scpep1	PRD34546	2	269.79	1,921.7	2.82–2.84
Serine carboxypeptidases	B7PJ32, B7PJ51, DAA34155,	14	538.04	4,083.83	2.87–3.09
Serine protease K12H4.7	XP_003742341	1	2.03	18.12	3.15
Venom serine carboxypeptidase	XP_017788931, XP_026806100	2	6.68	75.55	3.15–3.60
Cysteine proteases					
Midgut cysteine proteinase 4	AAO60047	1	8.17	17.33	1.08

For each protein description, the accession codes and number of transcripts assigned, the average expression level in RPKM for unfed (RPKM 0) and 7 days fed (RPMK 7) females, and the range of log2FC reached are provided

Metalloproteases are proteolytic enzymes that require a metal ion, usually Zn^2+^, for their catalytic activity. They are present throughout the evolutionary scale, from bacteria to mammals, and are important components of snake venoms, where many of them act as haemorrhagic agents [95, 96]. In ticks, metalloproteases have been found in the midgut, ovary, salivary glands and saliva, where they are particularly abundant and diverse [45, 86, 97]. Tick salivary metalloproteases play functions related to blood-feeding and modulation of the host defensive responses, including degradation of extracellular matrix proteins at the bite site to form the feeding pool and degradation of fibrinogen and fibrin, thereby preventing blood coagulation, degradation of inflammatory mediators and inhibition of host tissue repair via anti-angiogenic activity [41, 92, 97–99]. Not surprisingly, metalloproteases were the enzyme class most abundantly represented in the *O. erraticus* sialotranscriptome (338 transcripts) (Table 4), in parallel with that also reported for other tick sialomes [29, 39, 41, 43, 93, 98]. These *O. erraticus* metalloprotease orthologues represent a wide repertory of enzymes, some of which have been functionally characterised. For instance, the enzymes known as “A disintegrin and metalloproteinase with thrombospondin motifs” (ADAMs), the peptidase M18B domain-containing protein and the endothelin-converting enzyme act as vasodilators and blood pressure regulators [100, 101]; metis metalloproteases act as fibrinolytic anti-clotting agents and as inhibitors of wound healing [102]; neprilysins may regulate host inflammatory and immune responses [103]. Since these functions may facilitate blood-feeding, some tick orthologues of these metalloproteases have been even tested as antigens for anti-tick vaccines [102, 104].

Serine proteases are also usual members of the argasid and ixodid sialomes. They are supposed to facilitate blood-feeding by regulating a range of host defensive mechanisms, including matrix remodelling, innate immunity and inflammation, blood clotting and fibrinolysis [45, 85, 93, 105, 106]. We found up to 47 serine protease transcripts in the upregulated sialotranscriptome of *O. erraticus* females (Table 4), suggesting their involvement in tick feeding.

Only one cysteine protease was identified in the upregulated sialotranscriptome of *O. erraticus*, the midgut cysteine proteinase 4 (AAO60047), which is related to cathepsin L of *Rhipicephalus* spp. ticks [107]. Cathepsin L is involved in blood digestion in ticks and was found upregulated after feeding in the midgut of *O. erraticus* [32]. There is no information on the potential function of cathepsin L in tick saliva during blood ingestion, but a recent study showed that a cathepsin L from *Rhipicephalus microplus* impairs thrombin-induced fibrinogen clotting via a fibrinogenolytic activity contributing to maintaining fluidity of ingested blood [108]. Reasonably, if this anti-clotting mechanism were present in tick saliva, it would also contribute to maintaining blood fluidity, thus facilitating its ingestion.

#### Protease inhibitors

Most of the host defensive responses that ticks must overcome to obtain a blood meal, including haemostasis, inflammation and immunity, are mediated by proteases (predominantly by serine and cysteine proteases) [104, 108]. Thus, to evade host defences and facilitate blood-feeding, tick sialomes contain abundant protease inhibitors, mainly serine and cysteine protease inhibitors [109, 110].

Tick serine protease inhibitors are classified in four groups: Kunitz domain inhibitors, Kazal domain inhibitors, trypsin inhibitor-like cysteine rich domain (TIL) inhibitors and serpins. Kunitz inhibitors are the most abundant in tick sialomes and form a large family of secreted anti-haemostatic proteins that inhibit various proteases in the coagulation cascade, primarily thrombin and activated factor X (FXa) [45, 111]. Serpins are also abundant in tick sialomes, where they act as suppressors of the host immune system and inhibitors of platelet aggregation and blood coagulation [83, 109]. The family of TIL-domain inhibitors includes chymotrypsin, elastase and trypsin inhibitors, which are ubiquitous in blood-feeding insects and tick sialomes. Some members of this family are known to interfere with the host inflammatory response and others have been characterised as antimicrobial peptides [99]. Kazal domain inhibitors are less frequent in tick sialomes and their function remains unknown [45, 109].

Tick cysteine protease inhibitors, or cystatins, are of two types. Type 1 cystatins are intracellular and participate in the intracellular digestion of haemoglobin and in developmental processes, while type 2 cystatins are mostly secreted into the host with saliva, where they act as immunomodulators [109].

There were 114 upregulated transcripts of protease inhibitors in the *O. erraticus* sialotranscriptome at 7 days after feeding (Table 5; Fig. 6a). Kunitz-domain inhibitors were largely the most abundant ones, as they totalled 86 transcripts, including savignygrin and papilin-like proteins. Savignygrin is a disintegrin-type inhibitor of platelet aggregation first identified from *O. kalaharensis* [112]; recently, a savignygrin orthologue has been found in the sialome of *O. moubata* [43]. Papilins are multi-Kunitz-domain proteins of the extracellular matrix involved in basement membrane formation and embryonic development [113, 114]. They have been recently found in the sialome of *O. rostratus* and *O. moubata*, but its function in tick saliva remains to be established [41, 43].

**Table 5:** Protease inhibitors differentially upregulated 7 days after feeding (7 vs 0)

Description	Accession	No. transcripts	RPKM 0	RPKM 7	Log_2_FC
Kunitz domain-containing proteins					
Carboxypeptidase inhibitor SmCI-like isoform X2	XP_022249736	1	5.601	55.65	3.31
Chymotrypsin inhibitor	BAM28739, ACF57858, XP_020288964, XP_019890240	7	128.53	290.81	1.10–1.27
CLUMA_CG020329, isoform A	CRL07350	1	26.10	63.20	1.28
Dual kunitz salivary protein	ABR23474, ACB70326, ACB70328, ACB70330	24	963.30	10,876.82	1.71–5.17
Hypothetical protein B4U80_08130	RWS20793	1	1.28	4.07	1.67
Kunitz domain	ABI52641	2	14.11	45.86	1.69–1.71
Kunitz domain-containing salivary protein	ABR23431	14	1666.76	5958.42	1.70–1.94
Kunitz-type serine protease inhibitor	XP_023347625, XP_023347625, PRD26526, ODN00404	4	204.65	823.26	1.98–2.05
Papilin-like	XP_022252622, XP_023226201, XP_011307577, XP_026316323, XP_026316271	7	365.94	1573.38	2.05–2.14
Savignygrin	AAM54047, AAM54048	14	164.70	789.52	2.15–2.44
Serine protease inhibitor	B7PWG9, B7PG23, B7Q3H2	11	96.72	537.99	2.45–2.58
TIL-domain inhibitors					
Chymotrypsin-elastase inhibitor	P83516	2	21.52	217.19	3.33–3.34
Ixodidin	ACB70299	4	486.49	6598.35	3.35–4.24
Riddle, putative (Fragment)	B7QLA0	3	67.50	1349.76	4.25–4.48
Trypsin inhibitor-like	XP_028178942.1	1	2.26	51.32	4.51
Other protease inhibitors					
Alpha-2-macroglobulin	AAN10129, XP_023243497	14	421.09	3730.10	2.60–3.29
Cystatin	AZB52851	1	27.13	55.61	1.03
Kazal-type serine protease inhibitor domain protein	DAA34653	2	57.08	118.07	1.04–1.05
Pregnancy zone protein-like	XP_022257127	1	1.74	17.44	3.32

For each protein description, the accession codes and number of transcripts assigned, average expression level in RPKM for unfed (RPKM 0) and 7-day fed (RPMK 7) females, and the range of log_2_FC reached are provided

The remaining non-Kunitz protease inhibitors upregulated in the *O. erraticus* sialotranscriptome included several TIL-domain inhibitors, alpha-2 macroglobulin (α2M), one Kazal-domain inhibitor and one cystatin. Among the TIL-domain inhibitors found, ixodidin is remarkable because of its high upregulation (log_2_FC 3.35–4.24) and high expression level (6598.35 RPKM). It was first purified from the haemocytes of *Rhipicephalus microplus* and characterised as an antimicrobial peptide [115], suggesting a similar function in the saliva of *O. erraticus*. Alpha-2 macroglobulin belongs to an evolutionarily conserved family of universal protease inhibitors mainly involved in innate immunity against undesired proteolytic attacks. α2M is being increasingly detected in tick sialomes [29, 85], but only a few studies have dealt with the involvement of α2M in tick-feeding physiology. Two former studies showed that it participates in the immune defence of soft and hard ticks against microbes [116, 117]; more recently, Saravanan et al. [118] and Oleaga et al. [43] reported that α2M is expressed in the salivary glands of *O. moubata* and its expression is upregulated upon a blood meal feeding, in parallel with our current results. Given that human α2M has been observed to act as an anticoagulant by complexing substantial amounts of free thrombin [119], these authors suggest that, in addition to the functions of α2M in tick immunity, it could play an important function as an anticoagulant in saliva. The finding of high amounts of α2M in the saliva of *Amblyomma americanum* throughout the feeding period lends additional support to this hypothesis [29].

#### PLA2

The PLA2 constitute a superfamily of phospholipid-hydrolysing enzymes that includes a large family of secreted PLA2s. This family has up to 16 subgroups and includes the secreted PLA2s from animal venoms and tick saliva [45, 105, 120, 121]. Individual secreted PLA2s show unique tissue and cellular distributions and enzymatic specificities, justifying their varied biological activities that include toxic actions, virucidal and bactericidal activity, platelet aggregation inhibition, anticoagulation, regulation of host inflammation and immune response as well as novel ways for cellular signalling and membrane trafficking that do not depend on lipid mediators [120, 122–124].

Secreted PLA2s are present in the sialomes of soft and hard ticks [41, 43], but very few studies have dealt with their functional characterisation. A PLA2 from *O. moubata* salivary glands was shown to act as an antagonist ligand for host P-selecting, inhibiting the haemostatic and pro-inflammatory processes subsequent to expression of P-selecting on the damaged vascular endothelium of the host [125, 126]. More recently, PLA2 mRNA was found highly upregulated 7 days after feeding in the *O. moubata* sialotranscriptome, suggesting the importance of the antihaemostatic and anti-inflammatory functions exhibited by this protein in tick feeding [43]. Indeed, animal immunisation with a recombinant form of *O. moubata* PLA2 induced significant protection (44%) against *O. moubata* feeding, confirming the involvement of PLA2 in the feeding process and its utility as candidate antigen for anti-tick vaccine development [27].

In the *O. erraticus* sialotranscriptome, we detected 176 highly upregulated (log_2_FC 1.05–4.13) transcripts of PLA2 representing up to 7.2% (26,568.99 RPKM) of total expression at 7 days after feeding (Table 6; Fig. 6a). These figures represent a 14-fold expression rate and 8-fold expression level higher than those observed for PLA2 in the sialotranscriptome of *O. moubata* at 7 days after feeding [43]. This strongly suggests that PLA2 plays also an important role in the *O. erraticus* feeding process, highlighting and extending its potential utility as protective antigen for development of vaccines against *Ornithodoros* ticks.

**Table 6:** Acid and basic tail proteins, basic tailless, phospholipases A2 and heme-binding proteins differentially upregulated 7 days after feeding (7 *vs* 0)

Description	Accession	No. transcripts	RPKM 0	RPKM 7	Log_2_FC
Phospholipases A2					
Phospholipase A2	ABR23453, AGJ90343, ISCW020728-PA, ACB70350, ABI52736 ABI52805, AWU67138	176	3574.67	26,568.99	1.05–4.13
Heme-binding proteins					
Apolipoprotein B-100, partial	KFM77310	1	8.67	17.63	1.02
GP80 precursor, partial	AAA92143	1	21.77	543.30	4.64
Hemelipoglyco-carrier protein	AJR36491	4	140.84	327.54	1.18–1.34
Hemelipoglycoprotein precursor	ABD83654	1	103.13	317.73	1.62
Vitellogenin	B7QJ67, BAH02666, AAW78557	8	196.58	4404.67	3.42–4.99
Vitellogenin-1	ALC78840, ASB34115	2	23.69	432.64	3.83–4.25
Acid tail proteins					
Acid tail salivary protein	ABR23355, ACB70371, ABR23361, ACB70369, ABR23412	46	6985.634	30,613.23	1.16–3.28
Basic tail proteins					
Basic tail salivary protein	ABR23429, ABR23472, ACB70313	8	1090.89	4346.36	1.12–2.47
BTSP	ABI52639, ACB70365, ACB70321, ABI52632, ACB70394	21	1156.79	4931.17	1.49–3.38
Basic tailless proteins					
Salivary basic tailless protein	ABR23379	13	5590.63	15,443.41	1.18–1.83

For each protein description, the accession codes and number of transcripts assigned, the average expression level in RPKM for unfed (RPKM 0) and 7-day fed (RPMK 7) females, and the range of log2FC reached are provided

#### Heme-binding proteins

The iron-containing heme group is a functional component of many haemoproteins and is required for normal tick physiology. Since ticks are unable to synthesise heme, they must obtain it from the blood meal. However, since free heme is toxic to tick cells, its distribution, storage and detoxification must be tightly regulated [127, 128]. Heme-binding proteins, including hemelipoglyco-carrier protein (CP) and vitellogenins (Vgs), participate in the removal and detoxification of free heme excess. Additionally, they participate in lipid transport and storage, and vitellogenins are precursors of vitellin, which is critical for egg development and oviposition [122–130]. Tick CP and Vgs share a similar evolutionary origin and structure, greatly complicating their differentiation, tissue expression and function assignments. Vgs were thought to be synthesised in the midgut and fat body and to be absent from salivary glands, where heme transport and storage would be dependent on the CP [129–131]. However, Vg mRNA has recently been found in the salivary glands of *Rhipicephalus bursa* [25] and *O. moubata* [43], where it is differentially overexpressed in response to blood-feeding; additionally, Vg protein has been found in the saliva of *A. americanum* [29] and *O. moubata* [85]. This proves that Vgs are also synthesised in the salivary glands and secreted into the host with tick saliva, which suggests that these proteins could serve different functions during tick feeding. Since the free heme group has proinflammatory activity, it has been proposed that heme-binding proteins secreted to saliva could reduce the concentration of free heme at the feeding lesion, in turn reducing its cytotoxicity and potential to promote inflammation [25, 29]. Additionally, tick saliva hemelipoproteins have been suggested to function as antioxidants and transporters of cholesterol, phospholipids and fatty acids [25, 132]. Indeed, RNAi gene knockdown of *R. bursa* salivary vitellogenin-3 significantly increased tick mortality after feeding, supposedly as a consequence of increased cellular toxicity and unbalanced energy production owing to compromised lipid storage [25].

We found 17 upregulated transcripts of heme-binding, Vg-like proteins in the sialotranscriptome of *O. erraticus*, representing 1.6% of the total mRNA expression 7 days after feeding (Table 6; Fig. 6a), which is consistent with that observed in other tick sialomes [29, 43]. This suggests that Vg-like proteins are abundantly synthesised in the salivary glands of *O. erraticus* and secreted into the host with saliva where they most likely play a relevant role in tick feeding. These findings, together with the recognised high immunogenicity of tick Vgs [25], make Vg-like proteins promising candidate antigens for tick vaccines.

#### Basic tail/tailless and acid tail proteins

Basic tail proteins are a tick-specific protein superfamily of hundreds of members and are so named because most of them have a highly basic carboxy terminus, usually composed of lysine and arginine residues. On the contrary, some family members have an acidic tail, mainly composed of glutamate, and are thus named acid tail proteins; other members of this superfamily lack the tail and are appropriately named basic tailless proteins [86, 105]. These proteins are ubiquitous and abundantly found in the sialotranscriptomes of ixodid and argasid ticks, which suggest that they play important and specific roles at the tick-host feeding interface [41, 43, 45, 82, 86, 105]. However, only a few members of this family have been functionally characterised as anticoagulants [133–135] and specific complement inhibitors [136], while the remaining members have as yet unknown functions.

Up to 46 acid tail, 29 basic tail and 13 basic tailless upregulated transcripts were identified in the sialotranscriptome of *O. erraticus*, representing 8.3%, 2.5% and 4.2%, respectively, of total mRNA expression at 7 days after feeding (Table 6; Fig. 6a). These results parallel those previously published on the expression of basic tail superfamily members in other argasid and ixodid ticks [41, 82] and suggest an important role for these molecules in the *O. erra*ticus feeding process, which deserves further investigation, as these proteins may constitute attractive targets to drug and/or immune interventions. From the perspective of finding target antigens for tick vaccine development, these tick-specific proteins would have the additional advantage that they do not share any homology to host proteins and might not cross-react with the host.

### Relevance of the current research for public and animal health

As stated in the background section, *O. erraticus* is a medical and veterinary concern because it transmits several *Borrelia* spp. spirochetes that cause TBRF as well as the virus that cause the ASF. This is a highly contagious and lethal disease in domestic pigs without effective treatment or vaccine, which causes dramatic economic impact in the affected regions [6, 7]. In the Mediterranean Basin, *O. erraticus* colonises anthropic environments and lives in close association with swine on free-range pig farms, hidden inside and around pig premises, which contributes to the transmission and persistence of ASF and TBRF in affected areas [5, 137]. On the other hand, ASF has recently spread out of control from the Caucasus to China, where there is a suspicion that local tick species belonging to the *O. erraticus* complex might be also competent vectors of the virus [10–13]. This possibility would have an enormous veterinary and economic relevance since it would significantly contribute to the increase of spread, transmission and persistence of ASF throughout that area, greatly complicating its prevention and control. Accordingly, any strategy aimed to be effective at the control of ASF and TBRF will require the elimination of *O. erraticus* populations (and maybe of other *O. erraticus-*complex species) from at least the anthropic environments. For this endeavour, anti-tick vaccines have emerged as the most promising and sustainable control strategy alternative to application of chemical acaricides [19].

In the current research we have used transcriptomics to approach the identification of potentially protective candidate antigens for vaccine development. We have analysed and filtered omics data and selected several functional groups and families of tick salivary proteins predicted to have important functions in biological processes related to blood-feeding. These proteins include histamine-binding and moubatin-like lipocalins, 5'-nucleotidases/apyrases, several metalloproteases, protease inhibitors, soluble PLA2s, vitellogenin-like heme-binding proteins and basic/acid tail and tailless proteins. Most of these protein families are the same as those recently described from the *O. moubata* sialotranscriptome [43] and all together contribute to increase the currently scant repertory of potentially protective candidate antigens from argasid ticks.

These protein groups and families show high functional redundancy, as most of their members act as anticoagulants, anti-platelet aggregation agents or as modulators of host innate immunity and inflammation. This functional redundancy teaches us which are the host defensive responses that must be necessarily annulled by the ticks to be able to feed. Additionally, functional redundancy strongly suggests that vaccines targeting individual tick antigens will probably be insufficient to reach full protection against ticks (i.e., completely blocking tick feeding) and highlights the convenience of developing multiantigenic vaccines targeting functionally redundant tick antigens to abolish the involved tick anti-defensive mechanisms and achieve full protection.

This research provides a set of promising candidate antigens which may be useful for developing vaccines for the control of *O. erraticus* infestations and the prevention of tick-borne diseases of public and veterinary health relevance, such as TBRF and ASF.

Finally, it should be noted that although this work was devoted to explore the potential of the upregulated salivary transcripts as vaccine candidate antigens, this does not mean that the downregulated transcripts have been discarded as vaccine candidates. It can be assumed that the genes that are downregulated in response to feeding are likely playing functions directly involved in feeding or regulatory functions somehow related to feeding. Therefore, the transcripts that were highly overexpressed before feeding and then downregulated at 7 days after feeding (Additional file 8: Table S8) might also be interesting as vaccine candidates and, consequently, the subject of future studies.

## Conclusions

We have assembled *de novo* the sialotranscriptome of *O. erraticus* using next-generation sequencing technologies, resulting in 22,007 high-confidence transcripts clusters and 18,961 annotated transcripts, which represent 86.15% annotation success. These transcripts encode thousands of proteins, many of which belong to large multigene protein families that are conserved between argasid and ixodid ticks. These data significantly increase the number of protein-coding sequences from argasid salivary glands currently available in public databases.

The complexity and functional redundancy observed in the sialotranscriptome of *O. erraticus* are comparable to those observed in the sialomes of other soft and hard ticks, with lipocalins, metalloproteases, acid tail proteins, PLA2s and protease inhibitors being the protein families most abundantly expressed.

Differential gene expression analysis in salivary glands along the trophogonic cycle showed that most of the differentially upregulated gene expression takes place in the first 7 days after feeding, being less relevant from day 7 post-feeding onwards. This suggests that *O. erraticus* replace the salivary bioactive proteins consumed for feeding early in the off-host period to get ready for the following blood meal. Since *O. erraticus* and the argasid ticks typically are fast feeders, they do not need to change their saliva composition along the feeding process. This is different in ixodid ticks, which experience the so-called “sialome and saliva switching”, which adds an additional level of complexity to their sialomes.

Functional GO term and metabolic pathway enrichment analyses of the differentially upregulated genes after feeding revealed several overrepresented protein groups and families functionally involved in blood ingestion and regulation of the host defensive responses, including lipocalins, metalloproteases, protease inhibitors, secreted phospholipases A2, 5′-nucleotidases/apyrases and heme-binding proteins, all of which can be interesting candidate protective antigens for the development of vaccines against *O. erraticus.*

All these proteins display great functional redundancy showing the host defensive responses that must be necessarily abolished by *O. erraticus* to be able to feed. Functional redundancy emphasises the need to develop multiantigenic anti-tick vaccines that target functional orthologue antigens to completely abolish the involved tick anti-defensive mechanisms and therefore reach full protection.

This sialotranscriptome provides a valuable reference database of ongoing studies aimed at obtaining the proteomes of the salivary glands and saliva of *O. erraticus*, which will in turn validate this transcriptome assembly. The integration of these transcriptomic and proteomic data will drive selection of antigenic candidates for developing vaccines for the control of *O. erraticus* infestations, which may contribute to preventing tick-borne diseases of public and veterinary health significance, such as TBRF and ASF.

## Supplementary Information

**Additional file 1: Table S1.** Metrics for *de novo* assembled transcriptomes of each salivary gland sample, for consensus transcriptomes at 0, 7 and 14 days after feeding and for the merged transcriptome.

**Additional file 2: Table S2.** Annotated sialotranscriptome of *O. erraticus*.

**Additional file 3: Table S3.** Pfam domain occurrences in the *O. erraticus* predicted proteins. A total of 9828 Pfam domains were observed in the *O. erraticus* proteins, of which 2458 were unique. Up to 6619 of the proteins contained at least one Pfam domain. The top 30 Pfam domain ocurrences are highlighted.

**Additional file 4: Table S4.** Biological pathways represented in the transcriptome of *O. erraticus salivary* glands, enzymes and transcript sequences involved in each pathway. The analysis of the salivary annotated genes was done in the KEGG pathway database.

**Additional file 5: Table S5**. Differentially expressed genes (DEG) in the salivary glands of *O. erraticus* females between three physiological states: 7-day fed *vs* unfed (7 *vs* 0), 14-day fed *vs* 7-day fed (14 *vs* 7) and 14-day fed *vs* unfed (14 *vs* 0).

**Additional file 6: Table S6.** Significantly enriched GO terms assigned to the differentially expressed transcripts in the three comparisons: 7-day fed *vs* unfed (7 *vs* 0), 14-day fed *vs* 7-day fed (14 *vs* 0) and 14-day fed *vs* unfed (14 *vs* 0). NumInCat, total number of sequences in the category. NumDEInCat, number of enriched sequences in each category.

**Additional file 7: Table S7.** Significantly enriched pathways in the three comparisons: 7-day fed *vs* unfed (7 *vs* 0), 14-day fed *vs* 7-day fed (14 *vs* 0) and 14-day fed *vs* unfed (14 *vs* 0). NumInCat, total number of sequences in the category. NumDEInCat, number of enriched sequences in each category.

**Additional file 8: Table S8**. Differentially downregulated genes in the salivary glands of *O. erraticus* between unfed and 7-day fed females (7 *vs* 0). The top 100 transcripts most highly expressed at day 0 (in average RPKM) are highlighted in green. Average RPKM 0 and average RPKM 7, average expression level in RPKM of the two biological replicas of unfed and 7-day fed females, respectively.

## Data Availability

The transcriptome data generated for this study were deposited at the National Institute for Biotechnology Information (NCBI) under bioProject ID PRJNA666995 and biosample accessions SAMN16339901, SAMN16339902, SAMN16339903, SAMN16339904, SAMN16339905, SAMN16339906. This Transcriptome Shotgun Assembly project has been deposited at DDBJ/EMBL/GenBank under the accession GIXX00000000. The version described in this paper is the first version, GIXX01000000.

## Abbreviations

α2M: Alpha-2 macroglobulin
ASF: African swine fever
CP: Hemelipoglyco-carrier protein
FDR: False discovery rate
GO: Gene ontology
KEGG: Kyoto Encyclopedia of Genes and Genomes database
NCBInr: National Center for Biotechnology Information non-redundant
ORF: Open reading frame
PBS: Phosphate-buffered saline
RPKM: Read per kilobase per million reads
TBRF: Tick-borne human relapsing fever
Vg: Vitellogenins
